# Supplementary material for: Identification of photoperiod-induced specific miRNAs in the adrenal glands of Sunite sheep (Ovis aries)
Source: Front Vet Sci. 2022 Jul 22;9:888207. doi: 10.3389/fvets.2022.888207 (PMC9354845; doi:10.3389/fvets.2022.888207)
Supplement: Supplementary file 1 [file Data_Sheet_1.ZIP › Supplementary file/miRDeep2 file.zip!/miRDeep2 file/Novel_Structure/SPLP42A2/Novel_670.pdf]

Provisional ID : NC_019481.2_40320
Score total : 3159.3
Score for star read(s) : 3.9
Score for read counts : 3151.9
Score for mfe : 2
Score for randfold : 1.6
Score for cons. seed :
Total read count : 6194
Mature read count : 6129
Loop read count : 0
Star read count : 65

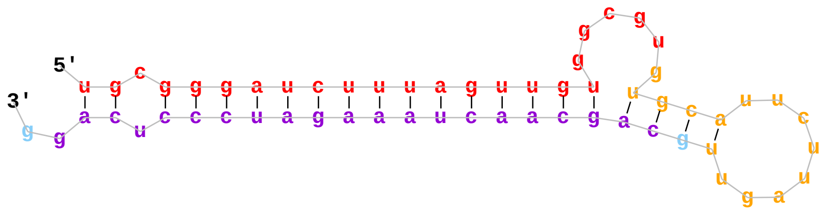

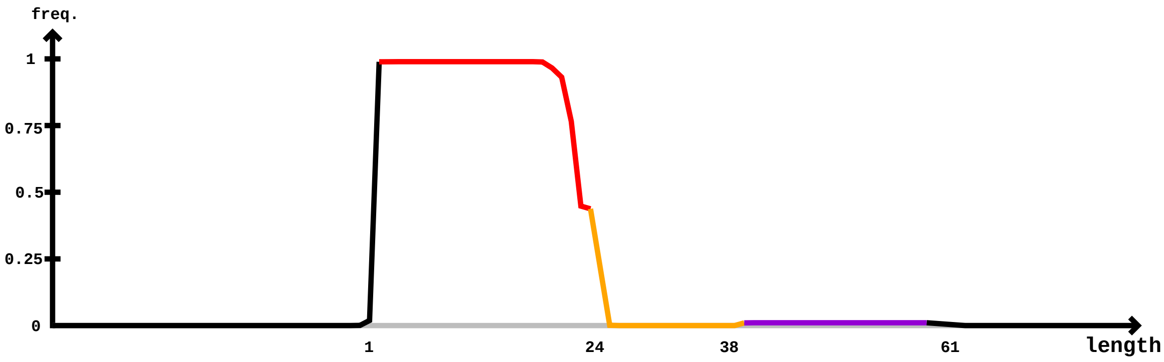


```
                              Mature                              Star
5'- aauaacccauaaaaagcagccgacguugcggcaugcgggaucuuuaguuguggcgugugcauucuuaguugcagcaacuaaagaucccucaggcugcaaugaagaucagaga -3'   obs
    aauaacccauaaaaagcagccgacguugcggcaugcgggaucuuuaguuguggcgugugcauucuuaguugcagcaacuaaagaucccucaggcugcaaugaagaucagaga         exp
    ....................(..(((((((((.((.(((((((((((((((......((((........))))))))))))))))))).)).)))))))))..)........   reads   mm   sample
    ...............................caugcgggaucuuuaguugug............................................................   1       0    seq
    ...............................caugcgggaucuuuaguuguA............................................................   1       1    seq
    ...............................caugcgggaucuuuaguuguggU..........................................................   1       1    seq
    ...............................caugcgggaucuuuaguuguggc..........................................................   1       0    seq
    ................................augcgggaucuuuaguugu.............................................................   10      0    seq
    ................................augcgggaucuuuaguuguA............................................................   2       1    seq
    ................................augcgggaucuuuaguugug............................................................   14      0    seq
    ................................augAgggaucuuuaguugug............................................................   1       1    seq
    ................................augcgggaucuuuaguugugA...........................................................   9       1    seq
    ................................augcgggaucuuuaguugugU...........................................................   1       1    seq
    ................................augcgggaucuuuaguuguggU..........................................................   19      1    seq
    ................................augcgggaucuuuaguuguggc..........................................................   47      0    seq
    ................................augcgggaucuuuaguuguggcg.........................................................   10      0    seq
    ................................augcgggaucuuuagCuguggcgu........................................................   1       1    seq
    .................................ugcgggaucuuuaguuA..............................................................   5       1    seq
    .................................ugcgggaucuuuaguug..............................................................   2       0    seq
    .................................ugcgggaucuuuaguugC.............................................................   21      1    seq
    .................................ugcgggaucuuuaguugu.............................................................   99      0    seq
    .................................ugcgggaucuuuaguugA.............................................................   6       1    seq
    .................................ugcAggaucuuuaguugu.............................................................   3       1    seq
    .................................ugcgAgaucuuuaguugu.............................................................   1       1    seq
    .................................ugcgggaucuuuaguugug............................................................   150     0    seq
    .................................ugcgggaucuuuaguuguA............................................................   40      1    seq
    .................................ugUgggaucuuuaguugug............................................................   1       1    seq
    .................................ugcgAgaucuuuaguugug............................................................   1       1    seq
    .................................ugcgggaucuuuaguugCg............................................................   2       1    seq
    .................................ugcgggaucuuuaguuguU............................................................   54      1    seq
    .................................ugcAggaucuuuaguugugg...........................................................   4       1    seq
    .................................ugcgggaucuuuaguugugA...........................................................   224     1    seq
    .................................ugcgggaucuuuUguugugg...........................................................   1       1    seq
    .................................ugcgggaucuuuaguugugg...........................................................   718     0    seq
    .................................ugcgggaucuuuagCugugg...........................................................   1       1    seq
    .................................ugcgggaucuuuaguuguAg...........................................................   4       1    seq
```

```
                                Mature                                Star
aauaacccauaaaagcagccgacguugcggcaugcgggaucuuuaguuguggcgugugcauucuuaguugcagcaacuaaagaucccucaggcugcaaugaagaucagaga
................................ugcggAaucuuuagguugugg...........................................................  1     1  seq
................................ugcgggaucuuuaguugCgg...........................................................  5     1  seq
................................ugUgggaucuuuaguugugg...........................................................  4     1  seq
................................ugGgggaucuuuaguugugg...........................................................  1     1  seq
................................ugcgggaucuuuaguugugAc..........................................................  7     1  seq
................................ugcgggaucuuuagCuguggc..........................................................  1     1  seq
................................ugcgggaucuuuaguuguggc..........................................................  1355  0  seq
................................ugcgggaucuuuaguugAggc..........................................................  1     1  seq
................................ugcgggaucuuuaguuguAgc..........................................................  3     1  seq
................................ugcgggaucuuuaguuguggU..........................................................  519   1  seq
................................ugUgggaucuuuaguuguggc..........................................................  6     1  seq
................................ugcgggaucuuuaguugCggc..........................................................  4     1  seq
................................ugcgggaucuuuUguuguggc..........................................................  1     1  seq
................................ugGgggaucuuuaguuguggc..........................................................  1     1  seq
................................ugcgggaucuuuagCuguggcg.........................................................  10    1  seq
................................ugcgggGucuuuaguuguggcg.........................................................  20    1  seq
................................ugcgAgaucuuuaguuguggcg.........................................................  19    1  seq
................................ugcUggaucuuuaguuguggcg.........................................................  4     1  seq
................................ugcgggaucuuuagCuguggcgu........................................................  7     1  seq
................................ugcgggGucuuuaguuguggcgu........................................................  13    1  seq
................................ugcgggaucuuuaguuguggcgu........................................................  2680  0  seq
................................ugcUggaucuuuaguuguggcgu........................................................  1     1  seq
................................ugcAggaucuuuaguuguggcgug.......................................................  2     1  seq
................................ugcgggaucuuuaguuguggcgugu......................................................  6     0  seq
.................................gcgggaucuuuaguuguggU..........................................................  1     1  seq
..................................cgggaucuuuaguuguggcg.........................................................  1     0  seq
..................................cgggaucuuuaguuguggcgu........................................................  1     0  seq
......................................................................cagcaacuaaagaucccuca.....................  21    0  seq
......................................................................cagcaacuaaagaucccucag....................  41    0  seq
.......................................................................agcaacuaaagaucccucag....................  1     0  seq
.......................................................................agcaacuaaagaucccucaggc..................  2     0  seq
```
